# Brain MRI segmentation of Zika-Exposed normocephalic infants shows smaller amygdala volumes

**DOI:** 10.1371/journal.pone.0289227

**Published:** 2023-07-28

**Authors:** Shanchita Ghosh, Teddy Salan, Jessica Riotti, Amrutha Ramachandran, Ivan A. Gonzalez, Emmalee S. Bandstra, Fiama L. Reyes, Samita S. Andreansky, Varan Govind, Gaurav Saigal

**Affiliations:** 1 Department of Radiology, University of California Davis, Sacramento, California, United States of America; 2 Department of Radiology, Miller School of Medicine, University of Miami, Miami, Florida, United States of America; 3 Department of Radiology, Jackson Memorial Hospital, Miami, Florida, United States of America; 4 Department of Radiology and Biomedical Imaging, University of California San Francisco, San Francisco, California, United States of America; 5 Division of Pediatric Infectious Diseases, Department of Pediatrics, Miller School of Medicine, University of Miami, Miami, Florida, United States of America; 6 Division of Neonatology, Department of Pediatrics, Miller School of Medicine, University of Miami, Miami, Florida, United States of America; 7 Sylvester Comprehensive Cancer Center, Miller School of Medicine, University of Miami, Miami, Florida, United States of America; Faculdade Sao Leopoldo Mandic, BRAZIL

## Abstract

**Background:**

Infants with congenital Zika syndrome (CZS) are known to exhibit characteristic brain abnormalities. However, the brain anatomy of Zika virus (ZIKV)-exposed infants, born to ZIKV-positive pregnant mothers, who have normal-appearing head characteristics at birth, has not been evaluated in detail. The aim of this prospective study is, therefore, to compare the cortical and subcortical brain structural volume measures of ZIKV-exposed normocephalic infants to age-matched healthy controls.

**Methods and findings:**

We acquired T2-MRI of the whole brain of 18 ZIKV-exposed infants and 8 normal controls on a 3T MRI scanner. The MR images were auto-segmented into eight tissue types and anatomical regions including the white matter, cortical grey matter, deep nuclear grey matter, corticospinal fluid, amygdala, hippocampus, cerebellum, and brainstem. We determined the volumes of these regions and calculated the total intracranial volume (TICV) and head circumference (HC). We compared these measurements between the two groups, controlling for infant age at scan, by first comparing results for all subjects in each group and secondly performing a subgroup analysis for subjects below 8 weeks of postnatal age at scan. ZIKV-exposed infants demonstrated a significant decrease in amygdala volume compared to the control group in both the group and subgroup comparisons (p<0.05, corrected for multiple comparisons using FDR). No significant volume differences were observed in TICV, HC, or any specific brain tissue structures or regions. Study limitations include small sample size, which was due to abrupt cessation of extramural funding as the ZIKV epidemic waned.

**Conclusion:**

ZIKV-exposed infants exhibited smaller volumes in the amygdala, a brain region primarily involved in emotional and behavioral processing. This brain MRI finding may lead to poorer behavioral outcomes and warrants long-term monitoring of pediatric cases of infants with gestational exposure to Zika virus as well as other neurotropic viruses.

## Introduction

Although the Zika virus (ZIKV) epidemic has subsided markedly, the impact of congenital ZIKV infection on the developing brain of children who were prenatally infected is still not completely understood. In infants with congenital Zika syndrome (CZS), microcephaly and other macro-anatomical brain abnormalities have been confirmed using ultrasound, CT, and conventional MR imaging [1–8]. However, a larger proportion of infants who were prenatally exposed to ZIKV (ZIKV-exposed), due to maternal ZIKV infection during pregnancy, are normocephalic at birth without apparent clinical or macro-anatomical brain abnormalities [9]. Emerging reports suggest that these ZIKV-exposed infants may develop diminished head circumference months after birth [10]. A recently published report indicates that 32% of ZIKV-exposed children, up to age 3, have below-average neurological development [11]. Extensive brain malformations and neurological abnormalities are seen in these children, which are thought to be secondary to microstructural damage caused by ZIKV on the developing fetal brain [11].

The aims of this prospective study were (a) to examine the radiological manifestations in infants exposed to maternal ZIKV infection who demonstrate no microcephaly or other gross macroanatomical abnormalities, (b) to quantify specific brain tissue type volumes in ZIKV-exposed and matched control infants, and (c) to make between-group comparisons. Analysis of the brain imaging measures will provide a baseline for longitudinal neuroimaging assessments in this population.

## Materials and methods

### Subjects

This prospective study is HIPAA-compliant and was approved by the Institutional Review Board of the University of Miami Miller School of Medicine and the Jackson Health System, Miami, Florida. Both ZIKV-exposed and healthy control infants were recruited from the Jackson Memorial/Women’s Hospital and Holtz Children’s Hospital services, Miami, Florida. Parents of all infants included in this study provided written informed consent.

For the purposes of this study, ZIKV *exposure* was defined as a positive ZIKV specific immunoglobin M (IgM) enzyme-linked immunosorbent assay (ELISA) result in maternal serum without microcephaly in the infant. Conventional plaque reduction neutralization testing (PRNT) was performed for the detection of Zika virus and Dengue virus. Infants with ZIKV *infection* were defined as infants with characteristic findings associated with CZS including microcephaly, gross ventriculomegaly, and parenchymal calcifications; such infants were excluded from this study. Microcephaly was defined as head circumference more than 2 standard deviations below the mean for gestational age and sex [12]. The inclusion criteria for the ZIKV-exposed infant group included mothers with confirmatory Zika testing by the Florida Department of Health (i.e., IgM-positive and confirmatory PRNT results). Infants whose mothers refused testing for Zika were excluded from the study. Infants whose mothers had other evidence of viral or parasitic infection such as HIV, syphilis, rubella, cytomegalovirus, and toxoplasmosis during pregnancy were also excluded.

A total of 49 infants were recruited for the study and 36 completed the MRI scans. Of the remaining subjects, 10 were excluded for being above 20 weeks postnatal age at scan to focus on young infants. The final study cohort for this report consists of 18 ZIKV-exposed and 8 healthy control infants. Mothers of the participating infants were screened at enrollment for major depression during pregnancy using the Edinburgh Postnatal Depression Scale (EPDS) and for use of anti-depressive medication.

### MR imaging and volumetric measurements

Infants were scanned without sedation and during natural sleep time on a 3T MRI scanner (General Electric) using a 32-channel head coil. They were restrained on a papoose board and monitored with EKG and pulse oximeter during the scan. High-resolution axial T2-weighted MR images of the brain were obtained using a T2-PROPELLER sequence (TE: 68.7 ms; TR: 13.6 s; slice thickness: 2 mm; in-plane spatial resolution: 0.625 x 0.625 mm^2^; voxel volume: 0.625 x 0.625 x 2 = 0.78 mm^3^, acquisition time = 2 minutes 15 seconds). The use of this sequence has helped to reduce the effect of subject motion and magnetic susceptibility artifacts in images as the infants were scanned without sedation. The protocol also included axial susceptibility weighted imaging, proton MR spectroscopy, diffusion-weighted imaging, T1-weighted FLAIR, and T1-weighted BRAVO sequences for a total acquisition time of approximately 25 minutes.

**Fig 1:**
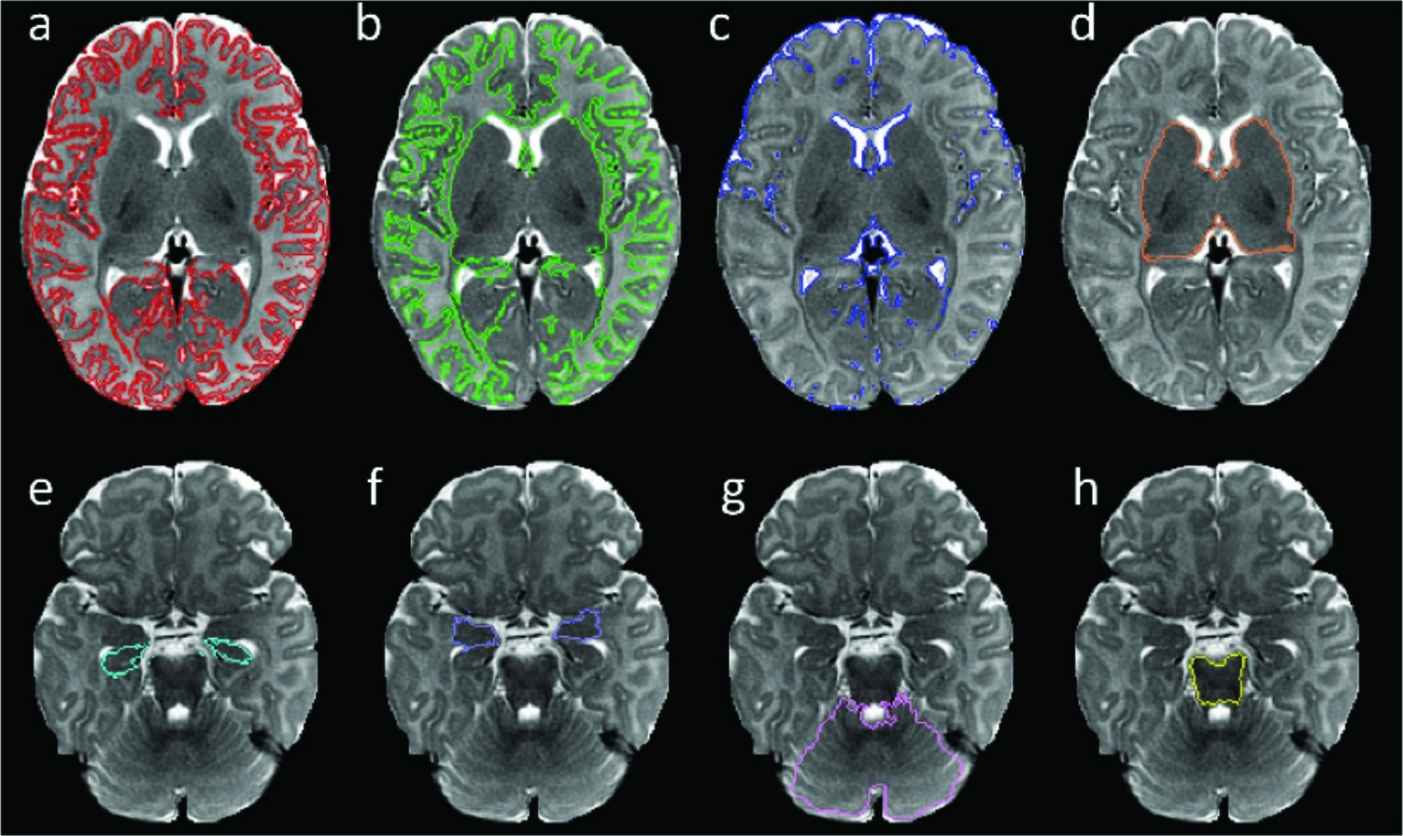
Representative axial slices of a T2-weighted MR image obtained from a ZIKV-exposed subject. The colored contours highlight the 8 ROIs segmented by MANTiS as: (a) cortical grey matter in red, (b) white matter in green, (c) cerebrospinal fluid in dark blue, (d) nuclear deep grey matter in orange, (e) hippocampus in light blue, (f) amygdala in purple, (g) cerebellum in pink, and (h) brainstem in yellow.

Brain images were processed using the Morphologically Adaptive Neonate Tissue Segmentation (MANTiS) program [13]. MANTiS is a tissue segmentation toolbox developed for the Statistical Parametric Mapping (SPM) software. It segments T2-weighted brain images of infants using adaptive templates into 8 different tissue types defined as distinct anatomical regions of interest (ROI) and include white matter (WM), cortical grey matter (GM), deep nuclear GM, corticospinal fluid (CSF), amygdala, hippocampus, brainstem, and cerebellum (Fig 1). Radiologists with at least three years of experience reviewed all images for quality and assessed these diagnostically. The radiologist co-authors reviewed the ROIs segmented by the MANTiS for accuracy of the tissue types (i.e., grey matter or white matter) or anatomical delineations of each ROI and manually edited segmented ROIs as needed. The most performed manual edits were for removal of erroneously segmented extracranial soft tissues and incomplete segmentation of the cerebellum. We noted incomplete imaging of the posterior fossa in two subjects that were excluded from the calculation of cerebellum and brainstem volumes. We calculated the volumes of all eight regions from the segmentation results by multiplying each ROI’s voxel count by the voxel size. For each subject, we calculated the total intracranial volume (TICV) by adding the volumes of all eight segmented ROIs. In order to reduce the effects of variability due to individual head size differences, we normalized each subject’s ROI volumes by dividing them by TICV. As such, TICV is reported in absolute volume (cm^3^), while the other measurements are reported as normalized volumes as a percentage of TICV between 0 and 1.

### Statistical analysis

Between-group differences in demographics and EPDS scores were checked using Wilcoxon and Chi-squared tests for continuous and categorical variables, respectively. If any of the gestational age, age at scan, or postmenstrual age were found to be significantly different, these variables were controlled for as covariates in the analysis of head circumference and the 9 brain volumetric measurements (8 ROIs + TICV). Measurement outcomes were first checked for normality of residuals with Shapiro-Wilk tests, and for homogeneity of residual variance with Levene’s tests. We also tested for homogeneity of regression slopes to determine if there were significant interactions between the covariate and the grouping variable. If those conditions were met, between-group comparisons were performed using analysis of covariance (ANCOVA) at 95% confidence level (p < 0.05) corrected for multiple comparisons using false-discovery rate (FDR) [14]. Otherwise, if assumptions of normality and homogeneity were not met, non-parametric ANCOVA procedures were used instead. Finally, a correlation analysis was performed between the neuroimaging measurements and EPDS scores to find associations between the depression level of mothers and the brain developmental outcomes of their infants. All statistical tests were performed using the R programming language.

## Results

Demographic information for the ZIKV-exposed and healthy control groups are provided in Table 1A. Infants from both groups were matched for gestational age (GA), with full-term pregnancies for all controls, with only 3 ZIKV-exposed infants born preterm at 35 and 36 weeks GA and were not significantly premature to exclude from the study. The two groups did differ in their age at scan with slightly older ZIKV-exposed babies. This, however, did not impact the overall postmenstrual age (PA) which was not significantly different. Both groups were also matched for term gestation, sex, and race with subjects primarily from black or white Hispanics ethnic backgrounds. Mothers of ZIKV-Exposed infants did not exhibit signs of higher depression during pregnancy than mothers of controls, with only one mother in each group showing major depression (EPDS > 13) and only one mother of a ZIKV-Exposed infant was taking anti-depressive medication.

**Table 1:** Demographic characteristics of ZIKV-Exposed and control infants, and the Edinburgh Postnatal Depression Scale (EPDS) of their mothers.

**A: Complete Cohort**	ZIKV-Exposed	Control	*p*
Subject count	18	8	
Gestational age (weeks; mean ± SD)	38.4 ± 1.6	39.1 ± 0.9	0.34
Age at scan (weeks; mean ± SD)	8.7 ± 4.8	5.3 ± 2.6	0.04
Postmenstrual age (weeks; mean ± SD)	47 ± 5	44.4 ± 3	0.17
Gestation (percentage and count)			0.22
Term	83.3% (15)	100% (8)	
Pre-term	16.7% (3)	0% (0)	
Infant Sex (percentage and count)			0.48
Male	50% (9)	37.5% (3)	
Female	50% (9)	62.5% (5)	
Infant Race/Ethnicity (percentage and count)			0.55
White Hispanic	55.6% (10)	37.5% (3)	
Black Hispanic	5.6% (1)	0% (0)	
Black	38.8% (7)	62.5% (5)	
EPDS of mothers (mean ± SD)	4.13 ± 4.73	6.8 ± 4.2	0.2
EPDS > 13 (count)	1	1	
**B: Subgroup (<8 weeks age at scan)**	ZIKV-Exposed	Control	*p*
Subject count	9	7	
Age at scan (weeks; mean ± SD)	5.11 ± 1.5	4.5 ± 1.3	0.29
Gestational age (weeks; mean ± SD)	38.6 ± 1.8	39.1 ± 1	0.79
Postmenstrual age (weeks; mean ± SD)	43.7 ± 2	43.6 ± 2	0.67
Gestation (percentage and count)			0.36
Term	88.9% (8)	100% (7)	
Preterm	11.1% (1)	0% (0)	
Infant Sex (percentage and count)			0.95
Male	44.4% (4)	42.9% (3)	
Female	55.6% (5)	57.1% (4)	
Infant Race/Ethnicity (percentage and count)			0.64
White Hispanic	44.4% (4)	42.9% (3)	
Black Hispanic	11.1% (1)	0% (0)	
Black	44.4% (4)	57.1% (4)	
EPDS of mothers (mean ± SD)	4.43 ± 5.03	7.56 ± 3.81	0.2
EPDS > 13 (count)	1	0	

EPDS = Edinburgh Postnatal Depression Scale; SD = standard deviation

Since age at scan was the only variable that differed between ZIKV-exposed and control infants, we controlled for age at scan as a covariate in the subsequent analysis of head circumference and brain volumetric measures. We also performed a subgroup analysis by selecting subjects below 8 weeks of age at scan from both groups to obtain an age-matched sample. This resulted in 9 ZIKV-exposed and 7 controls who showed no significant differences in any of the demographic characteristics (Table 1B).

**Fig 2:**
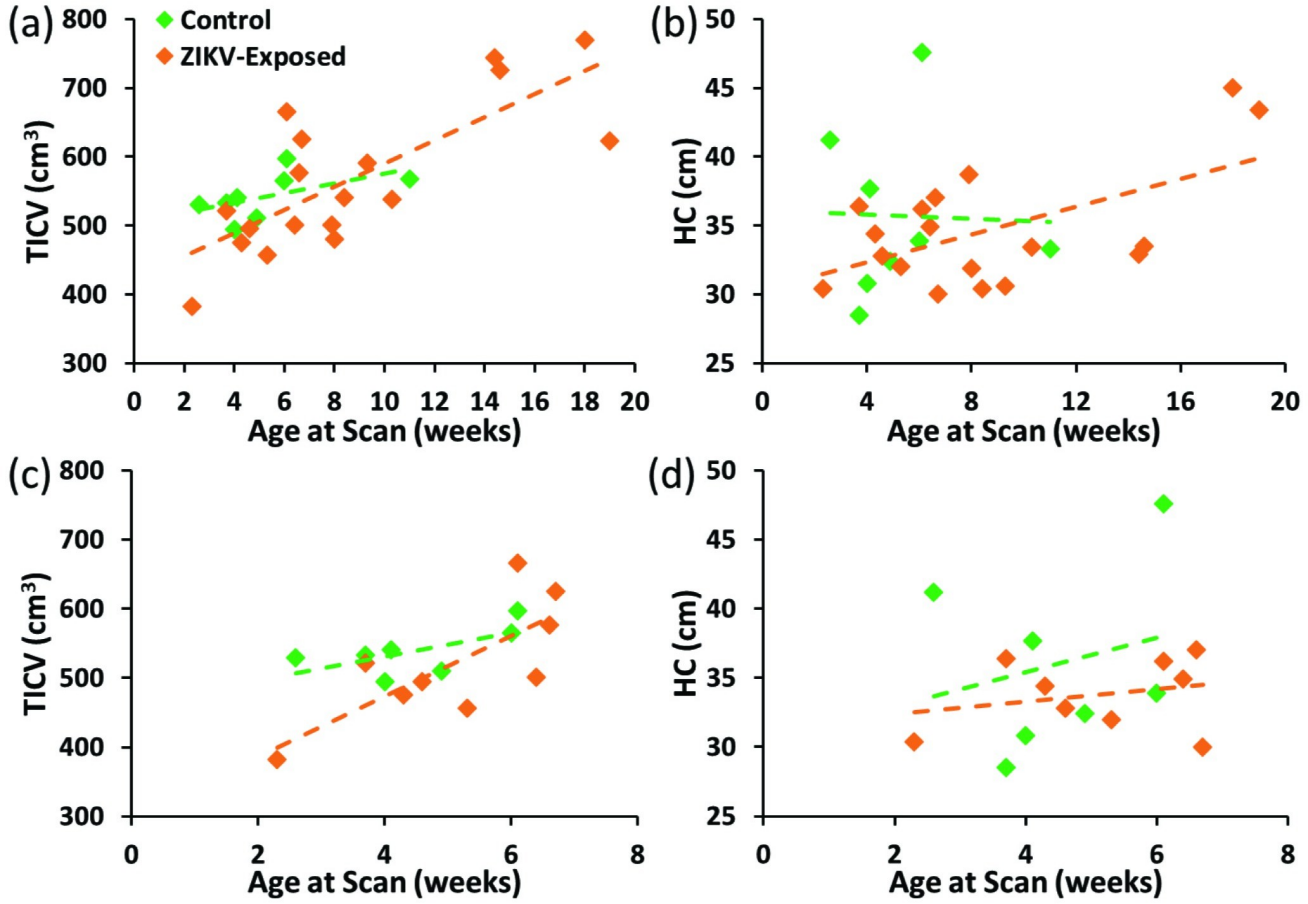
Scatter plots showing age at scan in weeks with respect to (a) total intracranial volume (TICV) and (b) head circumference (HC) for the complete cohort, and for the sub-group analysis with subjects below 8 weeks of age (c and d). Although the regression lines appear to be converging, the homogeneity of slopes was maintained in all cases showing that there was no significant interaction between age at scan and the grouping variable (p > 0.05). (TICV = total intracranial volume, HC = head circumference).

**Fig 3:**
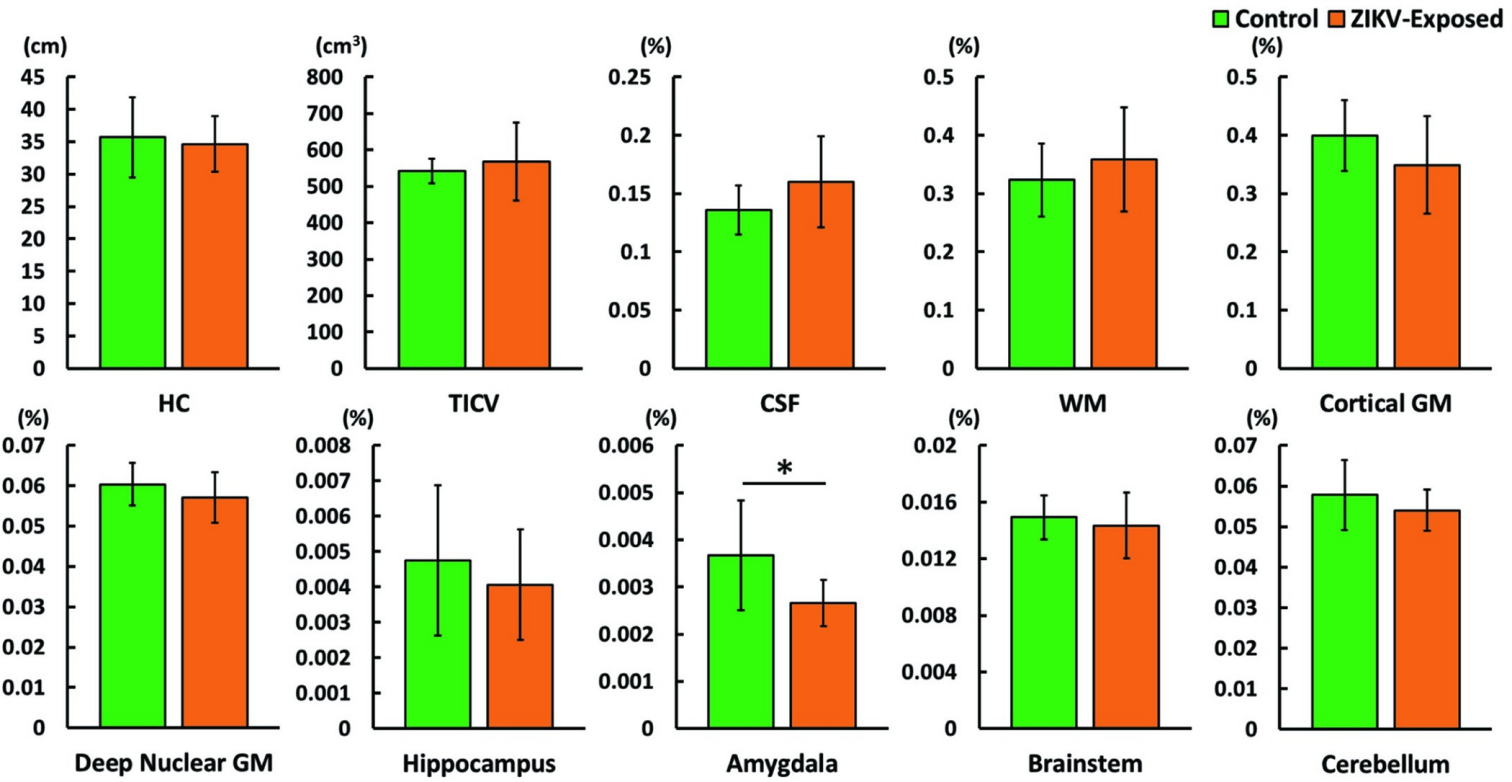
Comparisons of group mean and standard deviation between control and ZIKV-Exposed subjects for the following measurements: Head circumference (HC), total intracranial volume (TICV), and the normalized volumes of cerebrospinal fluid (CSF), white matter (WM), cortical grey matter (GM), deep nuclear GM, hippocampus, amygdala, brainstem, and cerebellum. * p < 0.05 (FDR corrected).

ANCOVA analysis showed no significant interactions (p > 0.05) between age at scan and the grouping variable (Fig 2), confirming that the homogeneity of slopes assumption was met, and the difference in age did not influence the outcome in measurements between controls and ZIKV-exposed subjects. We observed significant decreases in volume in the ZIKV-exposed group only in the amygdala (controls: 0.0036; ZIKV-exposed: 0.0027; p = 0.013). There were no appreciable differences in the overall size of the brain measured by TICV and HC, or in any other brain structures (Fig 3). The subgroup analysis showed identical results, with homogeneity of slopes (p > 0.05) when controlling for age (Fig 2), and between-group differences only observed in the amygdala (controls: 0.0038; ZIKV: 0.0026; p = 0.003) while other measures did not significantly differ. Results of the correlations between the EPDS scores and TICV, HC, and the remaining volumetric measures did not show significant associations between depression experienced by the mothers and the brain development of their infants.

## Discussion

To the best of our knowledge, this is the first study to segment brain T2-MRIs obtained from ZIKV-exposed infants into anatomical regions, component tissue types, and CSF. The main finding from this prospective case-control study is that there was a significant volume reduction in the amygdala for the ZIKV-exposed group when evaluating both the complete ZIKV-exposed cohort and its subgroup below 8 weeks of age. There is no published report in the literature on decreased amygdala volume or its underlying pathological causes in ZIKV-exposed human infants. We therefore looked for findings from animal model ZIKV infection studies [15, 16] as well as human studies [17–22] on associations between psychological distress or depression experienced by mothers during the pregnancy and the amygdala volume in infants born to them for corroboratively interpreting the results of this study. Longitudinal studies of postnatally ZIKV-infected infant macaques at 3, 6, and 12 months of age have shown smaller amygdala volumes and reduced functional connectivity in amygdala-connected brain networks, which regulate emotional behavior and functions [15, 16]. Furthermore, these changes to the amygdala in ZIKV-infected macaques corresponded with changes in their emotional responses to stress inducing stimuli [15, 16]. However, findings in the literature on associations between the amygdala volume of human infants and psychological stress experienced by their mothers during pregnancy were inconsistent [17–22]. Reports show lower amygdala volumes in male neonates [17] and 4 year old children [18, 19], but larger amygdala volumes in girls [19, 20], associated with higher prenatal maternal distress, depressive symptoms, and anxiety during pregnancy. Larger amygdala volumes and lower functional connectivity were also found in newborn infants of mothers who experienced prematernal distress due to Covid-19 infection [21], while investigation of the amygdala’s microstructure in neonates showed no relation to the mother’s anxiety [22]. Our results showed that mothers of ZIKV-exposed babies did not experience higher levels of depression during pregnancy, with only one mother in each group exhibiting major depression (EPDS > 13), and that higher EPDS scores did not correlate with smaller amygdala volumes or other measurements.

Since higher-level cognitive and functional deviations are often difficult to assess in infants, it is possible that specific disabilities will become more evident with increasing age. Several two to three-year follow-up studies of normocephalic ZIKV exposed infants, including a meta-analysis review that used data from 22 published articles, have indicated neurodevelopmental delays in language, cognition, and motor skills [23–25], deficits in the visual system [26], and low growth velocity with altered neurological and psychomotor development [27]. However, a small number of studies have also reported no risk of neurodevelopmental delays [28, 29]. Therefore, while most of the brain regions examined in our study do not appear to be abnormal in size, it is possible that structural abnormalities in ZIKV-exposed infants who were normocephalic at birth will become more evident at a later stage when neurodevelopmental delays will become more apparent. There is, in fact, a paucity of published neuroimaging studies with large sample sizes evaluating brain-imaging outcomes in this population beyond 3 years of age. A recent paper, which aggregated over 100,000 MRI scans to map growth trajectories of brain tissue types (i.e., cortical GM, WM, and deep GM) across the human lifespan, revealed that cortical GM growth peaked at nearly 6 years of age, with variability in cortical GM peaking at 4 years of age, while subcortical GM and WM peaked at 14.4 and 28.7 years of age, respectively [30]. These growth peaks in cortical GM were observed to be 2 to 3 years later than previously reported, which suggests that neuroanatomical developmental deficits in ZIKV-exposed infants may not manifest until at least 6 years of age. Furthermore, ZIKV exposed normocephalic newborns were found to have higher plasma concentrations of lysophosphatidylcholine relative to controls [31], which could contribute to a wide spectrum of clinical phenotypes such as neurodevelopmental, neurocognitive and ophthalmological abnormalities in childhood beyond microcephaly. Therefore, it is imperative to perform longitudinal neuroimaging and neurodevelopmental follow-ups of ZIKV-exposed infants to validate these early findings, but also to reassess the long-term clinical impact of ZIKV.

None of the remaining measurements we collected showed statistically significant between-group differences, although ZIKV-exposed infants appear to have higher CSF and WM volumes and concomitantly lower cortical and deep GM volumes. However, the significance of these observations is somewhat undermined by the sample size and the observed relatively higher standard deviations in the ZIKV-exposed group that reduced the statistical power of our study.

There are a few limitations to this study. As previously mentioned, first is the small sample size of ZIKV-exposed (n = 18) and control (n = 8) subjects. We were unable to enroll additional participants due to funding constraints. The waning ZIKV epidemic in our South Florida region and the nation led to an abrupt cessation of funding for this study by the Florida State Department of Health. Additionally, optimal assays and specimens used for testing, and the timing of testing for congenital ZIKV infection were unknown. The PRNT cannot distinguish between maternal and infant antibodies in specimens collected from infants at or near birth, and thus it was not performed on infants included in this study. Based on what is known about other congenital infections, maternal antibodies are expected to become undetectable by 18 months of age and might become undetectable earlier [32].

In summary, our study indicates that while overall brain volume measurements from MRIs performed on otherwise normal-appearing ZIKV-exposed infants indicate no significant change, a significant decrease in amygdala volume was still observed. Future longitudinal studies with a larger number of subjects and correlations with neurodevelopmental test scores would be needed to better understand the clinical and behavioral impact of ZIKV infection on pregnant mothers and ZIKV-exposure to their babies. If our results are reproduced in a larger study, then diagnostic evaluation and clinical management of these infants may warrant development of effective interventions.

## Supporting information

S1 Table Original data including age, EPDS scores, and brain measurements across all study participants. (XLSX)

## References

[1] de Fatima Vasco Aragao M, van der Linden V, Brainer-Lima AM, Coeli RR, Rocha MA, Sobral da Silva P, et al. Clinical features and neuroimaging (CT and MRI) findings in presumed Zika virus related congenital infection and microcephaly: retrospective case series study. BMJ. 2016;353:i1901. doi: 10.1136/bmj.i1901 27075009 PMC4830901

[2] Hazin AN, Poretti A, Di Cavalcanti Souza Cruz D, Tenorio M, van der Linden A, Pena LJ, et al. Computed Tomographic Findings in Microcephaly Associated with Zika Virus. N Engl J Med. 2016;374(22):2193–5. doi: 10.1056/NEJMc1603617 27050112

[3] Soares de Oliveira-Szejnfeld P, Levine D, Melo AS, Amorim MM, Batista AG, Chimelli L, et al. Congenital Brain Abnormalities and Zika Virus: What the Radiologist Can Expect to See Prenatally and Postnatally. Radiology. 2016;281(1):203–18. doi: 10.1148/radiol.2016161584 27552432

[4] Poretti A, Huisman T. Neuroimaging Findings in Congenital Zika Syndrome. AJNR Am J Neuroradiol. 2016;37(10):1764–5. doi: 10.3174/ajnr.A4924 27492074 PMC7960458

[5] de Souza AS, de Oliveira-Szjenfeld PS, de Oliveira Melo AS, de Souza LAM, Batista AGM, Tovar-Moll F. Imaging findings in congenital Zika virus infection syndrome: an update. Childs Nerv Syst. 2018;34(1):85–93. doi: 10.1007/s00381-017-3637-1 29181810

[6] Ribeiro BNF, Muniz BC, Gasparetto EL, Ventura N, Marchiori E. Congenital Zika syndrome and neuroimaging findings: what do we know so far? Radiol Bras. 2017;50(5):314–22. doi: 10.1590/0100-3984.2017.0098 29085165 PMC5656072

[7] de Fatima Viana Vasco Aragao M, de Lima Petribu NC, van der Linden V, Valenca MM, de Brito CAA, Parizel PM. Updated Imaging Findings in Congenital Zika Syndrome: A Disease Story That is Still Being Written. Top Magn Reson Imaging. 2019;28(1):1–14. doi: 10.1097/RMR.0000000000000193 30817674

[8] Schaub B, Gueneret M, Jolivet E, Decatrelle V, Yazza S, Gueye H, et al. Ultrasound imaging for identification of cerebral damage in congenital Zika virus syndrome: a case series. Lancet Child Adolesc Health. 2017;1(1):45–55. doi: 10.1016/S2352-4642(17)30001-9 30169227

[9] Honein MA, Dawson AL, Petersen EE, Jones AM, Lee EH, Yazdy MM, et al. Birth Defects Among Fetuses and Infants of US Women With Evidence of Possible Zika Virus Infection During Pregnancy. JAMA. 2017;317(1):59–68. doi: 10.1001/jama.2016.19006 27960197

[10] Reynolds MR, Jones AM, Petersen EE, Lee EH, Rice ME, Bingham A, et al. Vital Signs: Update on Zika Virus-Associated Birth Defects and Evaluation of All U.S. Infants with Congenital Zika Virus Exposure—U.S. Zika Pregnancy Registry, 2016. MMWR Morb Mortal Wkly Rep. 2017;66(13):366–73. doi: 10.15585/mmwr.mm6613e1 28384133 PMC5657905

[11] Nielsen-Saines K, Brasil P, Kerin T, Vasconcelos Z, Gabaglia CR, Damasceno L, et al. Delayed childhood neurodevelopment and neurosensory alterations in the second year of life in a prospective cohort of ZIKV-exposed children. Nat Med. 2019;25(8):1213–7. doi: 10.1038/s41591-019-0496-1 31285631 PMC6689256

[12] Hanzlik E, Gigante J. Microcephaly. Children (Basel). 2017;4(6). doi: 10.3390/children4060047 28598357 PMC5483622

[13] Beare RJ, Chen J, Kelly CE, Alexopoulos D, Smyser CD, Rogers CE, et al. Neonatal Brain Tissue Classification with Morphological Adaptation and Unified Segmentation. Front Neuroinform. 2016;10:12. doi: 10.3389/fninf.2016.00012 27065840 PMC4809890

[14] Chumbley JR, Friston KJ. False discovery rate revisited: FDR and topological inference using Gaussian random fields. Neuroimage. 2009;44(1):62–70. doi: 10.1016/j.neuroimage.2008.05.021 18603449

[15] Mavigner M, Raper J, Kovacs-Balint Z, Gumber S, O’Neal JT, Bhaumik SK, et al. Postnatal Zika virus infection is associated with persistent abnormalities in brain structure, function, and behavior in infant macaques. Sci Transl Med. 2018;10(435). 10.1126/scitranslmed.aao6975 PMC6186170 29618564

[16] Raper J, Kovacs-Balint Z, Mavigner M, Gumber S, Burke MW, Habib J, et al. Long-term alterations in brain and behavior after postnatal Zika virus infection in infant macaques. Nat Commun. 2020;11(1):2534. doi: 10.1038/s41467-020-16320-7 32439858 PMC7242369

[17] Lehtola SJ, Tuulari JJ, Scheinin NM, Karlsson L, Parkkola R, Merisaari H, et al. Newborn amygdalar volumes are associated with maternal prenatal psychological distress in a sex-dependent way. Neuroimage Clin. 2020;28:102380. doi: 10.1016/j.nicl.2020.102380 32805677 PMC7453059

[18] Acosta H, Tuulari JJ, Scheinin NM, Hashempour N, Rajasilta O, Lavonius TI, et al. Prenatal maternal depressive symptoms are associated with smaller amygdalar volumes of four-year-old children. Psychiatry Res Neuroimaging. 2020;304:111153. doi: 10.1016/j.pscychresns.2020.111153 32771833

[19] Acosta H, Tuulari JJ, Scheinin NM, Hashempour N, Rajasilta O, Lavonius TI, et al. Maternal Pregnancy-Related Anxiety Is Associated With Sexually Dimorphic Alterations in Amygdala Volume in 4-Year-Old Children. Front Behav Neurosci. 2019;13:175. doi: 10.3389/fnbeh.2019.00175 31447658 PMC6691065

[20] Wen DJ, Poh JS, Ni SN, Chong YS, Chen H, Kwek K, et al. Influences of prenatal and postnatal maternal depression on amygdala volume and microstructure in young children. Transl Psychiatry. 2017;7(4):e1103. doi: 10.1038/tp.2017.74 28440816 PMC5416711

[21] Manning KY, Long X, Watts D, Tomfohr-Madsen L, Giesbrecht GF, Lebel C. Prenatal Maternal Distress During the COVID-19 Pandemic and Associations With Infant Brain Connectivity. Biol Psychiatry. 2022. doi: 10.1016/j.biopsych.2022.05.011 35871095 PMC9110020

[22] Rifkin-Graboi A, Meaney MJ, Chen H, Bai J, Hameed WB, Tint MT, et al. Antenatal maternal anxiety predicts variations in neural structures implicated in anxiety disorders in newborns. J Am Acad Child Adolesc Psychiatry. 2015;54(4):313–21 e2. doi: 10.1016/j.jaac.2015.01.013 25791148

[23] Faical AV, de Oliveira JC, Oliveira JVV, de Almeida BL, Agra IA, Alcantara LCJ, et al. Neurodevelopmental delay in normocephalic children with in utero exposure to Zika virus. BMJ Paediatr Open. 2019;3(1):e000486. doi: 10.1136/bmjpo-2019-000486 31338431 PMC6613842

[24] Marban-Castro E, Vazquez Guillamet LJ, Pantoja PE, Casellas A, Maxwell L, Mulkey SB, et al. Neurodevelopment in Normocephalic Children Exposed to Zika Virus in Utero with No Observable Defects at Birth: A Systematic Review with Meta-Analysis. Int J Environ Res Public Health. 2022;19(12). doi: 10.3390/ijerph19127319 35742566 PMC9223424

[25] Mulkey SB, Arroyave-Wessel M, Peyton C, Bulas DI, Fourzali Y, Jiang J, et al. Neurodevelopmental Abnormalities in Children With In Utero Zika Virus Exposure Without Congenital Zika Syndrome. JAMA Pediatr. 2020;174(3):269–76. doi: 10.1001/jamapediatrics.2019.5204 31904798 PMC6990858

[26] Blackmon K, Evans R, Fernandes M, Landon B, Noel T, Macpherson C, et al. Neurodevelopment in normocephalic children with and without prenatal Zika virus exposure. Arch Dis Child. 2022;107(3):244–50. doi: 10.1136/archdischild-2020-321031 34479857 PMC8857021

[27] Peixoto L, Abtibol-Bernardino MR, Guerra CVC, de Oliveira GA, Chaves BCS, de Souza Rodrigues C, et al. Growth Velocity and Nutritional Status in Children Exposed to Zika Virus during Pregnancy from Amazonas Cohort, Brazil. Viruses. 2023;15(3). doi: 10.3390/v15030662 36992371 PMC10056230

[28] Grant R, Flechelles O, Tressieres B, Dialo M, Elenga N, Mediamolle N, et al. In utero Zika virus exposure and neurodevelopment at 24 months in toddlers normocephalic at birth: a cohort study. BMC Med. 2021;19(1):12. 33472606 10.1186/s12916-020-01888-0 PMC7819189

[29] Sobral da Silva PF, Eickmann SH, Arraes de Alencar Ximenes R, Ramos Montarroyos U, de Carvalho Lima M, Turchi Martelli CM, et al. Pediatric neurodevelopment by prenatal Zika virus exposure: a cross-sectional study of the Microcephaly Epidemic Research Group Cohort. BMC Pediatr. 2020;20(1):472. doi: 10.1186/s12887-020-02331-2 33038931 PMC7547521

[30] Bethlehem RAI, Seidlitz J, White SR, Vogel JW, Anderson KM, Adamson C, et al. Brain charts for the human lifespan. Nature. 2022;604(7906):525–33. doi: 10.1038/s41586-022-04554-y 35388223 PMC9021021

[31] da Costa Faria NR, Chaves-Filho AB, Alcantara LCJ, de Siqueira IC, Calcagno JI, Miyamoto S, et al. Plasma lipidome profiling of newborns with antenatal exposure to Zika virus. PLoS Negl Trop Dis. 2021;15(4):e0009388. doi: 10.1371/journal.pntd.0009388 33930014 PMC8115770

[32] Adebanjo T, Godfred-Cato S, Viens L, Fischer M, Staples JE, Kuhnert-Tallman W, et al. Update: Interim Guidance for the Diagnosis, Evaluation, and Management of Infants with Possible Congenital Zika Virus Infection—United States, October 2017. MMWR Morb Mortal Wkly Rep. 2017;66(41):1089–99. doi: 10.15585/mmwr.mm6641a1 29049277 PMC5689094

